# Hair cortisol and psychiatric symptomatology in children; outcomes of group CBT

**DOI:** 10.1016/j.cpnec.2024.100263

**Published:** 2024-09-12

**Authors:** Sarianna T.A. Barron-Linnankoski, Hanna K. Raaska, Paula H. Reiterä, Marja R. Laasonen, Marko J. Elovainio

**Affiliations:** a Child Psychiatry, Children and Adolescents, New Children's Hospital, Pediatric Research Center, University of Helsinki and Helsinki University Hospital, Helsinki, Finland; b Department of Psychology, Faculty of Medicine, University of Helsinki, Helsinki, Finland; c The Social Insurance Institution of Finland, Helsinki, Finland; d Biostatistics Consulting, Department of Public Health, University of Helsinki and Helsinki University Hospital, Helsinki, Finland; e Logopedics, School of Humanities, Philosophical Faculty, University of Eastern Finland, Joensuu, Finland; f Department of Psychology/ Research Program Unit, Faculty of Medicine, University of Helsinki, Helsinki, Finland; g The Finnish Institute for Health and Welfare, Helsinki, Finland

**Keywords:** Hair cortisol concentration, Behavior symptoms, Sleep disturbance symptoms, Cognitive behavioral therapy, Children, Psychiatric disorders

## Abstract

The associations between hair cortisol concentration (HCC), a biomarker of chronic stress, and behavior and sleep disturbance symptoms have not been studied in children with psychiatric disorders. While cognitive behavioral therapy (CBT) has proven effective in treating psychiatric symptoms in children, its potential biological implications as determined by HCC have not been investigated. We explored associations between HCC, behavior and sleep disturbance symptoms, and different diagnostic groupings (depression/anxiety, ADHD, or other types of psychiatric disorders) in clinician-diagnosed 6-12-year-old children (n = 100) with mixed psychiatric disorders and comorbidities. In addition, we examined whether group CBT led to changes in HCC, behavior symptoms, and sleep disturbance symptoms and whether any fluctuations in HCC levels were associated with potential symptom change. We collected data on HCC, internalizing and externalizing symptoms (The Spence Children's Anxiety Self-Report, Child Behavior Checklist, and Teacher Report Form), and sleep disturbance symptoms (The Sleep Disturbance Scale for Children) at three time points (baseline, post-treatment, and seven-month follow-up). Baseline HCC was not associated with behavior or sleep disturbance symptoms, whereas behavior and sleep disturbance symptoms were mutually correlated. No changes in HCC levels were observed with group CBT. Moreover, potential variations in HCC levels over the course of the study did not appear to be associated with behavior symptom relief after group CBT. Our findings suggest that HCC may not be a methodologically relevant biomarker of behavior or sleep disturbance symptoms in children with diverse psychiatric disorders.

## Highlights

• Hair cortisol levels were not associated with behavior or sleep disturbance symptoms.
• Hair cortisol concentration was not associated with behavior change after Group CBT.

## 1 Introduction

Individual and group-based CBT has shown immediate and long-term effectiveness in the treatment of childhood internalizing and externalizing symptoms (e.g. Refs. [34,52]. There is also burgeoning data on the effectiveness of CBT, namely CBT for insomnia, in treating childhood sleep disturbance symptoms [45]. The effectiveness of CBT has been studied at the symptom level, but little is known about whether effects can also be found at the biological level (e.g. Refs. [4,30,38,54]. One of the biological mechanisms of childhood psychopathology has been suggested to be physiological processes associated with chronic stress, such as dysregulation of the hypothalamic-pituitary-adrenal (HPA) axis [35]. Research has indicated that psychosocial interventions may modify cortisol regulation in children suggesting the potential for biological plasticity and reparation of regulatory systems through intervention [57].

Increasing evidence from reviews and meta-analyses has indicated HPA dysregulation in children and adolescents with various psychiatric disorders, such as depression, anxiety, and ADHD, with different underlying mechanisms assumed [15,22,35,42,60,68]. While research evidence on HPA axis dysregulation in developmental studies is mainly based on salivary cortisol [35], hair cortisol concentration (HCC) is increasingly used as a biomarker of HPA activity. A recent systematic review and meta-analysis has provided evidence for its utility in assessing chronic stress in children [39]. Associations between childhood stress, internalizing and externalizing psychopathology, and sleep disturbances are recognized (e.g. Refs. [2,28,47], but there is scarce and conflicting data on the physiological links between HCC and behavior and sleep problems. Advancing our understanding of the potential biological mechanisms involved between cortisol regulation and behavior and sleep disturbance symptoms in children with psychiatric disorders is relevant for evaluating treatment effects and guiding treatment targeting.

Findings from several cross-sectional studies have provided mixed evidence of positive, negative, or no associations between HCC and internalizing behaviors (e.g. Refs. [26,27,33,44,66]. In addition, findings by Ref. [23] suggested a curvilinear relationship between HCC and internalizing symptoms. Results on cross-sectional associations between HCC and externalizing behaviors are also inconclusive (e.g. Refs. [26,33,50,66]. Furthermore, the limited data on HCC and childhood sleep disorders provide conflicting results [20,47]. Most findings on the relationship between HCC and behavior or sleep disturbance symptoms are based on non-clinical samples (e.g. Refs. [20,23,26,33,44,50]. Little is known about HPA functioning using HCC in pediatric psychiatric samples [11,24,40,51]. Further investigation is needed to explore whether changes in HCC may contribute to behavior and sleep disturbance symptom alleviation in this population sample.

To the authors’ knowledge, there is no prior data on the potential biological implications of CBT, as assessed with HCC, in children with psychopathology. We identified a few studies addressing how HCC may relate to psychosocial treatment in children exposed to high levels of stress [16,29,49]. Two studies from the same group [16,49] reported an average one-third reduction in HCC levels in war-effected, trauma-exposed adolescents in response to psychosocial treatment (an 8-week stress-attunement group intervention) relative to wait-listed controls. Interestingly, the findings showed that treatment led to differing HCC trajectories over the 11-month study period depending on within-individual cortisol regulation. While HCC levels increased in those with hyposecretion, they decreased in those with intermediate or hypersecretion, suggesting that psychosocial interventions may lead to a normalization of HCC levels [16]. In another study involving children with adverse childhood experiences [29], changes in HCC over a year differed between groups according to whether children received psychosocial treatment (Child-Parent Psychotherapy) or not. In this study, the mean HCC level remained unchanged in the group that received treatment, while it decreased in the group that did not receive treatment. Whereas no cross-sectional association between HCC and behavior problems was observed at baseline, an increase in HCC over time was associated with a decrease in behavior problems across both groups, suggesting that HCC may also serve as a biomarker of behavioral symptom improvement [29]. We also identified a study on healthy 7–8-year-old children, in which a decrease in HCC was reported following a mindfulness-based program promoting self-perception and awareness [14]. To the best of our knowledge, there is no previous research on whether any changes in HCC during CBT are associated with potential behavior or sleep-related outcomes in children with psychiatric disorders.

The present study aimed to investigate the relationship between HCC, a biomarker of chronic stress, and behavior and sleep disturbance symptoms in a clinically referred sample of children with a variety of psychiatric disorders, and potential changes in HCC and symptoms after group CBT (GCBT). Firstly, we explored cross-sectional associations between HCC, behavior symptoms, namely internalizing and externalizing symptoms, and sleep disturbance symptoms at the baseline of the study. Given prior research on HPA dysregulation in various psychiatric disorders [15,35,42,68], we also explored associations between HCC and different diagnostic groupings. Secondly, we investigated whether GCBT leads to changes in HCC levels and behavior and sleep disturbance symptoms. In addition to assessing immediate changes after GCBT, we also examined the persistence of potential changes at a 7-month follow-up. Thirdly, we investigated the effects of any alterations in HCC levels on potential behavior and sleep disturbance symptom change over the course of the study. Based on prior research (e.g. Refs. [2,28], it was assumed that behavior and sleep disturbance symptoms would be associated with each other. Furthermore, behavior symptom alleviation as an impact of GCBT, was expected.

The study employed the Finnish versions of a generic form of CBT, the FRIENDS program [[6], [7], [8]], as FRIENDS was a GCBT protocol applied at the Helsinki University Hospital (HUS) specialized child psychiatric outpatient clinics (outpatient clinics) for children with a range of psychiatric disorders. Multiple informants (child, parent, and teacher) were used to obtain a comprehensive evaluation of the behavior and/or sleep disturbance symptoms, providing information from different perspectives and settings [1,36]. A prior study by the same group [9] focused specifically on behavior symptoms and found GCBT effective in treating a similar clinical sample of children with psychiatric disorders. The present study offers novel data on the association of HCC with behavior and sleep disturbance symptoms in children with psychiatric disorders and provides new insights into whether GCBT may influence behavior and sleep disturbance symptoms through the potential malleability of HCC in this population sample.

## 2 Methods

### 2.1 Participants and procedures

Participants were recruited between 2016 and 2018 through the routine referral process of the HUS outpatient clinics. Services are free of charge and are offered to school-aged children (ages 6 to 12) who are referred to HUS outpatient clinics by general practitioners (e.g., at family counseling centers, school health services, or health centers). Children were referred to GCBT if they showed symptoms of anxiety or depression, displayed problems in their emotional and behavioral skills, had sufficient social and cognitive abilities to engage in group work, and if both children and their caregivers (parents) expressed motivation for treatment. Exclusion criteria were excessive motoric restlessness and excessive aggressive behavior due to potential disruption to the GCBT as participants were required to be able to participate in group tasks. The clinical and diagnostic examinations carried out by multidisciplinary teams led by physicians served as the basis for the referral and exclusion criteria. All diagnoses were determined by MD specialists at the HUS outpatient clinics according to the diagnostic criteria of the 10th revision of the International Statistical Classification of Diseases and Related Health Problems [67]. The diagnostic procedures included thorough clinical assessment and data collection of the child, and the use of the required structured diagnostic measures, such as the Attention Deficit/Hyperactivity Rating Scale [19] the Autism Diagnostic Interview, revised [55], and the Autism Diagnostic Observation Schedule, second edition [43].

**Fig. 1:**
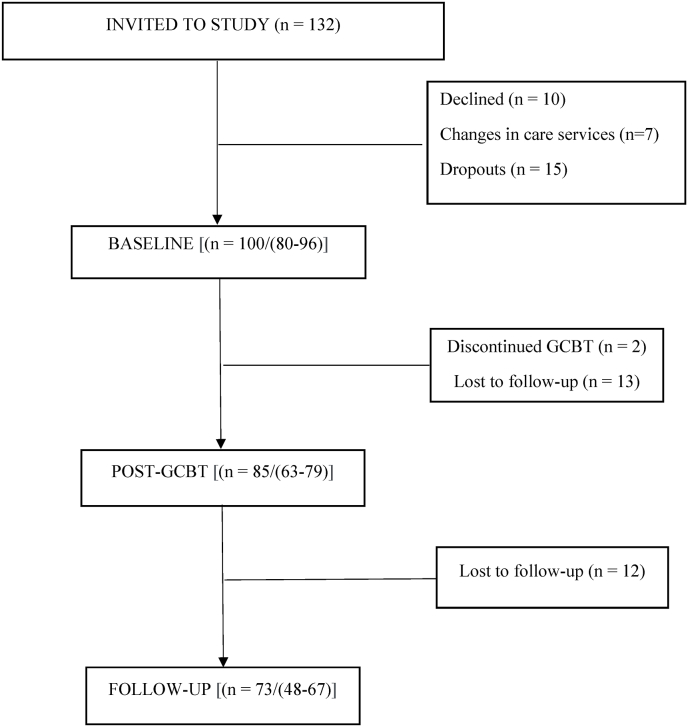
Study flowchart. Legend: From 132 subjects invited to the study 100 were enrolled. Outcome scores were analyzed at the following time points: baseline, post-GCBT, and at follow-up. The number (n) depicts participants/(returned outcome measures) at each time point.

All children (n = 132) referred for GCBT provided in outpatient clinics were invited to take part in the study. Children who were referred to GCBT, but whose severity of psychiatric symptoms required inpatient treatment (e.g., acute suicidality), were not included in the study since the GCBT protocol implemented in inpatient units was largely modified to suit those settings. After referral, informed written consent was obtained from the parents and children for participation in the study and the use of the measures. In addition, further informed written consent for the use of medical records in collecting data on the children's diagnoses, medication, family type, and school curriculum was received for 100 of the 132 children. Of all families invited to participate in the study, a final sample of 100 children (intention-to-treat sample) aged 6–12 years was enrolled (see Fig. 1 for flow chart). Further, informed written consent for the use of medical records for data collection was received for 94 of the 100 children. Demographic characteristics of the participants are presented in Table 1.

**Table 1:** Demographic data at baseline (n = 100).

Variable	n (%, Q_1-_Q_3_)
Gender	
Boys n (%)	68 (68.0)
Girls n (%)	32 (32.0)
Age in years, median (Q_1-_Q_3_)	9.9 (8.9–11.0)
Multiple diagnoses^a^ n (%)	70 (74.5)
ADHD^a^ n (%)	55 (58.5)
Depression or anxiety^a^ n (%)	35 (37.2)
Other disorders of psychological development^a^^,^^b^ n (%)	27 (28.7)
Conduct disorder^a^ n (%)	17 (18.1)
Autism spectrum disorder^a^ n (%)	16 (17.0)
Specific developmental disorders^a^^,^^c^ n (%)	15 (16.0)
Tic disorder^a^ n (%)	8 (8.5)
Maternal education level^d^ n (%)	
Low level	28 (28.9)
Mid-level	41 (42.3)
High level	28 (28.9)
Family type^a^ n (%)	
Nuclear family	48 (51.1)
Single-parent family	41 (43.6)
Adoption/foster care	5 (5.3)
Medication^e^	
Psychostimulants	44
Atomoxetine	4
Antipsychotics	4
Selective serotonin reuptake inhibitors	3

Note.

a Missing data for 6 %.

b Mainly difficulties with reciprocal social interaction.

c Diagnoses F80, F81 or F83 (ICD-10. 2016).

d Education level: low level = comprehensive or secondary education, mid-level = upper vocational education, high level = university education. Missing data for 3.0 %.

e Medication data for 100 of the 132 children invited to the study. Of these 100 children, 94 were included in the analyses of this study.

The GCBT treatment spanned a mean (M) of 4.7 months (standard deviation [SD] = 1.0), with 10 weekly group sessions and two booster sessions one and two months after session 10. Children who remained in HUS outpatient care during the 7-month follow-up period (M = 6.4 months, SD = 1.6 months after the return of post-GCBT measures; M = 7.5 months, SD = 1.6 months after the final GCBT session) had access to treatment-as-usual services at the HUS outpatient clinics after GCBT completion, if needed. These services were less intensive than the GCBT treatment consisting of individually tailored routine care, such as psychoeducation, supportive counseling, or assistance with family/school functioning from the child's clinical care manager (nurse, occupational therapist, psychologist, or social worker). Hair samples were collected, and data on behavior and sleep disturbance symptoms were gathered through self-, parent, and teacher reports at baseline, post-GCBT, and follow-up. Measures were returned either at an appointment or by post. The study was conducted in accordance with the Declaration of Helsinki and approved by the HUS Ethics Committee (49/13/03/03/2016 and HUS/2699/2018).

### 2.2 Treatment conditions

The GCBT treatment comprised three versions of the FRIENDS program depending on the age group of the participants: the Finnish translations of Fun FRIENDS [7] for children aged 6 to 8, FRIENDS for Life [6]for children aged 9–12, and My FRIENDS [8] for those turning 13 during the study. The choice of FRIENDS as the CBT-based intervention was related to the policies of HUS outpatient clinics, not to study-related factors. The FRIENDS program follows the core concepts of CBT, incorporating techniques and approaches that build an understanding of the connection between thoughts, feelings, and behavior. FRIENDS is an acronym that represents the themes covered in the program: F = Feelings; R = Relaxation; I = Inner thoughts; E = Exploring strategies; N = Now reward yourself; D = Don't forget to practice; S = Stay Calm. The treatment techniques of FRIENDS consist of psychoeducation, self-regulation, relaxation and mindfulness, empathy training, cognitive strategies, social support training, exposure, self-rewards, and relapse prevention. All versions of the FRIENDS program consisted of twelve 60-min sessions (ten weekly and two booster sessions). The FRIENDS program also included two group sessions for parents, which mostly focused on psychoeducation. However, due to clinic practices, a few groups (n = 5) did not receive the second booster session, and a few groups received just one (n = 6) or neither (n = 2) of the parent sessions.

A total of 31 groups were conducted at seven HUS outpatient clinics in the Helsinki metropolitan region area of Finland. Participants were divided into groups depending on their age and referral order. Each group had four to six participants and was led by two clinicians (nurses, occupational therapists, or psychologists). Nursing education in Finland is a bachelor's degree provided in universities of applied sciences. Following the standards of the FRIENDS program, all clinicians underwent a one-day FRIENDS training workshop. Sessions were carried out in accordance with the treatment manuals, with a focus on the key tasks depending on the children's pace in performing exercises.

Participants used the FRIENDS program workbooks throughout the treatment program and were given homework assignments, which however were not monitored in the study. Parents were asked to help their children with their homework and to continue using the workbook also after the end of the program.

### 2.3 Treatment integrity

The study design was presented and discussed at regular, recurring group leader meetings. All clinicians were kept informed of the study procedures and were contacted personally by email and/or telephone before the commencement of their group. Clinicians were encouraged to contact the research team for questions at any stage of the study. The clinicians notified the research team of the number of child and parent sessions attended by each family participating in the study.

### 2.4 Measures

#### 2.4.1 Hair cortisol concentration (HCC)

Hair samples, i.e. 2–3 hairlocks and about 20 individual pieces of hair strands, were cut from the back of the head, approximately 2 cm below the cranial bone, as close as possible to the scalp. The hair samples were wrapped in aluminum foil with a clear indication of the scalp end of the sample and stored at room temperature until sent to the Dresden LabService GmbH in Germany for analysis. Hair cortisol concentration (HCC) was assessed using liquid chromatography with coupled tandem mass spectrometry (LC-MS/MS) protocol [25]. The analysis was mainly performed on 2 cm long segments of the hair samples taken near the scalp, except for five post-GCBT hair samples and 17 follow-up hair samples for which analysis was performed on 3 cm long segments due to changes in laboratory procedures. The sample represented hormone secretion during the two to three-month period before hair sampling, based on an estimated average hair growth of 1 cm/month [65]. Results are reported as *picograms* per milligram (pg/mg).

#### 2.4.2 Behavior symptoms

The Child Behavior Checklist (CBCL), part of the Achenbach System of Empirically Based Assessment [2], is a parent-rated questionnaire on children's internalizing and externalizing symptoms. The CBCL consists of 113 items scored on a 3-point scale from 0 to 2 (0 = not true, 1 = somewhat/sometimes true, and 2 = very/often true). The CBCL comprises eight empirically based syndrome scales: 1) anxious/depressed, 2) withdrawn/depressed, 3) somatic complaints, 4) social problems, 5) thought problems, 6) attention problems, 7) rule-breaking behavior, and 8) aggressive behavior. Three of these scales (anxious/depressed, withdrawn, and somatic) form the internalizing (CBCL-Int) score, two (rule-breaking and aggressive behavior) form the externalizing (CBCL-Ext) score, and all eight scales form the total problem (CBCL-Tot) score. Scores below 60 reflect the normal range, scores from 60 to 63 the borderline range, and scores above 63 the clinical range. The CBCL has demonstrated excellent internal consistency for CBCL-Int, CBCL-Ext, and CBCL-Tot scores, and excellent test-retest reliability across the empirically-based problem scales [2]. The Finnish version has also shown high internal consistency for CBCL-Int and CBCL-Ext scores [31].

The Teacher's Report Form (TRF; [2]) is the teacher-rated counterpart of the CBCL, also comprising 113 items scored in the same way as the CBCL. The TRF has also demonstrated excellent internal consistency for the internalizing (TRF-Int), externalizing (TRF-Ext), and total problem (TRF-Tot) scores, and excellent test-retest reliability across the empirically-based problem scale [2]. The TRF was completed by the child's school teacher.

The Spence Children's Anxiety Self-Report (SCAS; [48]) is a self-report questionnaire assessing the severity of internalizing symptoms on six clinical subscales: (1) generalized anxiety, (2) panic/agoraphobia, (3) social phobia, (4) separation anxiety, (5) obsessive-compulsive disorder, and (6) physical injury fears. The SCAS comprises 44 items (38 items on symptoms of anxiety and 6 positive, filler items to reduce negative response bias) on a 4-point frequency scale from 0 to 3 (0 = never, 1 = sometimes, 2 = often, and 3 = always. The SCAS total and subscale scores are calculated by summing the scores for the relevant items. A *T-score* of 60 or higher is indicative of elevated anxiety. The SCAS has demonstrated very high internal consistency for total scores, acceptable internal consistency for subscales, and acceptable test-retest reliability for total scores over six months [58]. Furthermore, it has been found a reliable instrument for cross-cultural use [48]. If the child could not read, the caregiver read the questions aloud. The child was encouraged to respond according to their authentic experience.

#### 2.4.3 Sleep disturbance symptoms

The Sleep Disturbance Scale for Children [12] is a parent-rated questionnaire evaluating children's symptoms of sleep disorders in six different categories: (1) disorders of initiating and maintaining sleep, (2) sleep breathing disorders, (3) disorders of arousal/nightmares, (4) sleep-wake transition disorders, (5) disorders of excessive somnolence, and (6) sleep hyperhidrosis. The SDSC consists of 26 items on a Likert-type scale from 1 to 5 on specific sleep patterns (1 = never, 2 = occasionally, 3 = sometimes, 4 = often, 5 = always), and on an estimate of sleep quantity (e.g., 1 = 9–11 h and 5 = less than 5 h) and onset time (e.g., 1 = less than 15 min and 5 = more than 60 min). The sum of items provides a total score ranging from 26 to 130, with higher scores indicating greater sleep disturbance. A cutoff score of 39 has been suggested to identify children with sleep disturbance [12]. The SDSC has demonstrated good internal consistency in clinical samples of school-aged children, and adequate test-retest reliability [12].

### 2.5 Statistical analyses

Descriptive statistics are presented as frequencies and percentages (%), medians (Mdn) and inter-quartile ranges (Q_1_-Q_3_), or means (M) and standard deviations (SD) depending on variable distributions.

Outlying HCC values and symptom scores (CBCL, TRF, SCAS, and SDSC scores), and non-normal distributions were identified by graphical examination. Outliers were defined as rare but legitimate values and were therefore included in the analyses. The significance of outliers was assessed by Cook's distance in linear regression analyses.

Missing values mostly originated from unreturned measures at different time points rather than from treatment dropouts. No imputations were made to the HCC, or for CBCL and TRF measures which were scored using the ASEBA software. For SCAS, missing items in partially completed subscales were replaced using the mean of the completed items. Subscales were not scored if more than two items were missing, and the total score was considered missing if one or more subscales were missing. In total, 9, 19, and 5 imputations were made at baseline, post-GCBT and follow-up, respectively. For SDSC, missing values in partially completed questionnaires were manually substituted by individual total score means. Missingness was mainly due to parents not answering the first two items, most probably related to inattention, as they were slightly separated at the top of the questionnaire. The number of imputations for Q1 were 10, 13, and 8, and for Q2 were 15, 16, and 9 at baseline, post-GCBT, and follow-up, respectively. Furthermore, 14, 3, and 5 imputations were made for items 3–26 at baseline, post-GCBT, and follow-up, respectively. Missing scores were present mainly for one or two of the 26 items. At baseline, only 2 questionnaires had missing values for three items, and at follow-up only one questionnaire had three missing values (including items Q1 and Q2). Missing values other than for partially completed questionnaires were not imputed as this could distort the analyses of the relatively small data set and lead to incorrect conclusions by oversimplifying the variation in the data.

To investigate relationships between HCC and symptoms, we first computed Spearman's ranked correlation coefficients (ρ) among HCC, behavior (CBCL, TRF, and SCAS), and sleep disturbance symptom (SDSC) scores. We included demographic variables (age, gender, and maternal education) to account for possible confounding. As the HCC sample reflected hormone secretion from two to three months before hair sampling, we used HCC at two different time points (baseline and post-GCBT), while other variables were used only at baseline. Then, baseline associations of HCC with symptom scores, demographic variables, and diagnostic groups were examined using linear regression analysis. We formed three diagnostic groups: 1) participants diagnosed with depression or anxiety, 2) participants with ADHD, and 3) participants diagnosed with other diagnoses. These groupings were based on the diagnostic classifications in our study and research on HPA functioning in childhood psychiatric disorders (e.g. [15,35,68]. Due to skewness, HCC was log-transformed for the regression modeling. Maternal education was recoded into dummy variables for the regression analysis, with mid-level education (i.e. upper vocational education) chosen as the reference category. Multivariable regression models were adjusted for age and gender. Possible curvilinear associations of HCC with internalizing symptoms were assessed visually and by curvilinear regression analysis.

Secondly, we examined whether GCBT led to changes in HCC, behavior symptoms, and sleep disturbance symptoms across the three time points (baseline, post-GCBT, and follow-up) using Friedman tests, with pairwise comparisons performed using Dunn-Bonferroni tests. Since there was a larger amount of missingness for SDSC items Q1 and Q2 compared to the other SDSC items, we verified that the SDSC scores results did not differ whether these items were included in the analysis or not. Thirdly, a two-way mixed ANOVA was performed to analyze the effects of time (3 time points) and HCC on potential symptom change throughout the study. This analysis was performed only for the variables that showed a change between time points. The HCC variable was recoded as a categorical variable in terms of whether there was a decrease or increase in HCC levels from baseline to follow-up. Statistical analyses were performed using IBM SPSS Statistics versions 25.0 and 29.0. All analyses were undertaken using the Intent-to-Treat (ITT) principle. In all analyses, p-values <0.05 were considered statistically significant.

## 3 Results

### 3.1 Demographic descriptives

Demographic data for participants at baseline are shown in Table 1. There were no significant outliers as assessed by Cook's distance. Of the participants, 89.8 % were known to follow the national core curriculum for education (data missing for two participants). All participants for whom self- or parent ratings were obtained at post-GCBT and/or at follow-up (treatment completers n = 87), attended 7 or more GCBT sessions; 25.3 % attended 7–9 sessions and 74.7 % attended 10–12 sessions. Respectively, 29.9 % of the treatment completers’ parents attended zero parent sessions, 27.6 % one parent sessions, and 42.5 % two parent sessions.

### 3.2 Associations among variables

Bivariate correlations among variables are provided in Table 2A, Table 2B. The HCC values were negatively correlated with age at baseline but not at post-GCBT. In contrast, HCC correlated with gender post-GCBT but not at baseline. A Mann-Whitney *U* test showed a difference in HCC values between boys and girls at post-GCBT (*p* = .005) with boys presenting higher HCC (Mdn/IQR for boys: 7.30/3.07–21.59; for girls: 2.89/2.00–6.11). Baseline HCC was not correlated with symptom variables. Negative correlations were also observed between externalizing scores and age, and total behavioral problem scores and age. The CBCL total problem scores and the SCAS and SDSC total scores were all positively correlated. In addition, CBCL-Int and CBCL-Ext scores were positively correlated with SCAS total scores, and CBCL-Int scores with SDSC total scores. Furthermore, positive correlations were observed between CBCL-Int, CBCL-Ext, and CBCL-Tot scores, between CBCL-Ext and TRF-Ext scores, and between TRF-Int, TRF-Ext, and TRF-Tot scores. The p-values for correlations between variables were not Bonferroni-corrected.

**Table 2A:** Bivariate correlations among variables at baseline.

Variable	HCC	CBCL-Int	CBCL-Ext	CBCL-Tot	TRF-Int	TRF-Ext	TRF-Tot	SCAS	SDSC	Age	Gender	EDUM
HCC												
CBCL-Int	.209											
CBCL-Ext	.012	.334∗∗										
CBCL-Tot	.050	.699∗∗	.806∗∗									
TRF-Int	.044	.209	−.171	−.033								
TRF-Ext	.106	−.030	.322∗∗	.132	.410∗∗							
TRF-Tot	.105	.021	.135	.070	.653∗∗	.898∗∗						
SCAS	−.141	.390∗∗	.259∗	.352∗∗	.085	−.030	.012					
SDSC	.109	.391∗∗	.188	.375∗∗	.002	−.015	−056	.238∗				
Age	−.214∗	−.193	−.321∗∗	−.240∗	.027	−.149	−.087	−.140	−.090			
Gender	.185	.117	.161	.091	.181	.145	.111	−.210	.068	−.058		
EDUM	−.057	.163	.071	.133	−.129	−.083	−.110	.095	.173	−.040	−.029	

Note.

∗ p < .05 and.

∗∗ p < .01.

**Table 2B:** Bivariate correlations between post-GCBT hair cortisol concentration and baseline behavior and sleep disturbance symptoms, age, gender, and maternal education.

Variable	CBCL-Int	CBCL-Ext	CBCL-Tot	TRF-Int	TRF-Ext	TRF-Tot	SCAS	SDSC	Age	Gender	EDUM
HCC	.045	−.052	−.090	.169	−.028	.023	−.227	−.008	−.064	.326∗∗	−.026

Note.

∗∗ p < .01.

Multivariable linear regression did not find HCC values to be associated with symptom scores (CBCL, TRF, SCAS, and SDSC scores), demographic variables (age, gender, and maternal education), or any of the diagnostic groupings (depression/anxiety, ADHD, and other diagnoses) at baseline. No associations were detected in multivariable regression models (Table 3). The relationship between HCC and internalizing symptoms was not found to be curvilinear for self- or parent ratings (SCAS total score *B* = −.018, *p* = .526; SCAS total score^2^ *B* < .001, *p* = .760; CBCL-Int *B* = .103, *p* = .558; CBCL-Int^2^ *B* = −.001, *p* = .659). Although significance was observed in teacher ratings (TRF-Int *B* = −.025, *p* = .142; Trf-Int^2^ *B* = .001, *p* = .040), visual inspection did not show a curvilinear relationship.

**Table 3:** Multivariable regression associations between HCC and symptom variables.

Predictors	*B*	CI	*R*^2^_adj_	*p*
Model 1: CBCL-Int	0.023	−0.01 to 0.06	.022	.17
Model 2: CBCL-Ext	−0.009	−0.04 to 0.03	0.001	.61
Model 3: CBCL-Tot	0.005	−0.04 to 0.05	0.001	.82
Model 4: TRF-Int	0.024	−0.02 to 0.06	0.031	.26
Model 5: TRF-Ext	0.001	−0.04 to 0.04	0.012	.95
Model 6: TRF-Tot	0.016	−0.03 to 0.06	0.021	.44
Model 7: SCAS	−0.008	−0.03 to 0.01	0.012	.45
Model 8: SDSC	0.016	−0.02 to 0.05	0.017	.38
Model 9:			.016	
Dep/Anx	−0.555	−1.26 to 0.15		.123
ADHD	−0.253	−0.99 to 0.48		.495
Other dg	−0.169	−0.93 to 0.59		.658

Note: In each model HCC is the dependent variable. Models were adjusted for age and gender. Abbreviations: *B,* unstandardized beta*,* CI, confidence interval, *R*^2^_adj,_ adjusted R squared, *p* statistical significance.

### 3.3 Treatment outcomes across time points

Friedman's tests showed a difference between time points for the CBCL Int (χ2(2) = 15.055, *p* = .001), CBCL Ext (χ2(2) = 23.836, *p* **<** .001), and CBCL Tot scores (χ2(2) = 23.902, *p* **<** .001, n = 59 for all CBCL scores). Post hoc tests revealed decreases in CBCL-Int, CBCL-Ext, and CBCL-Tot scores from pre-to post-GCBT (*p* = .001 for CBCL-Int and *p* < .001 for CBCL-Ext and CBCL-Tot), and in CBCL-Ext and CBCL-Tot scores from baseline to follow-up (*p* = .001 and *p* = .004, respectively; unadjusted significance for CBCL-Int .053). In the total sample (see Table 4), the CBCL-Int scores were reduced from the borderline range to the normal range (Mdn change 4.0), the CBCL-Ext scores from the clinical range to the borderline range (Mdn change 3.5), and the CBCL Tot scores from the clinical range to the normal range (Mdn change 5.0) during GCBT. The CBCL-Ext and CBCL-Tot scores decreased from the clinical range to the borderline range (Mdn changes 3.5 and 3.0, respectively) from baseline to follow-up.

**Table 4:** Descriptive statistics of the outcome measures at the different time points.

Outcome measure	Baseline		Post-GCBT		Follow-Up	
	n	Mdn (Q1-Q3) Min-Max	n	Mdn (Q1-Q3) Min-Max	n	Mdn (Q1-Q3) Min-Max
HCC	85	5.33 (3.56–12.29) 0.39–1714.50	76	4.77 (2.45–13.66) 0.55–3240.00	59	7.60 (3.52–18.85) 0.95–331.76
CBCL						
CBCL-Int	92	62.50 (54.00–69.75) 41-86	78	58.50 (50.00–64.25) 34-79	67	61.00 (52.00–67.00) 39-77
CBCL-Ext	92	63.50 (56.50–70.00) 34-81	78	60.00 (51.00–65.00) 34-83	67	60.00 (51.00–65.00) 33-77
CBCL-Tot	92	64.00 (58.25–71.00) 45-77	78	59.00 (53.00–65.00) 38-79	67	61.00 (54.00–66.00) 43-77
TRF						
TRF-Int	80	64.00 (58.00–68.75) 42-90	63	63.00 (56.00–68.00) 42-89	48	63.50 (58.25–69.75) 42-84
TRF-Ext	80	64.50 (57.00–71.00) 42-79	63	63.00 (53.00–70.00) 42-84	48	59.50 (53.00–69.75) 42-85
TRF-Tot	80	67.00 (59.25–72.00) 50-90	63	65.00 (57.00–70.00) 37-81	48	64.50 (59.00–70.75) 39-82
SCAS						
Separation anxiety	88	3.00 (1.00–5.00)	78	3.00 (1.00–5.00)	63	3.00 (1.00–5.00)
Social phobia	88	3.00 (1.00–6.00)	78	2.00 (1.00–4.25)	63	3.00 (1.00–5.00)
Obsessive compulsive	87	4.00 (1.50–7.00)	77	3.00 (2.00–6.00)	63	3.00 (1.00–4.00)
Panic/agoraphobia	87	2.00 (1.00–5.00)	77	1.00 (0.00–4.00)	63	1.00 (0.00–3.00)
Physical injury fears	88	3.00 (1.63–6.00)	77	3.00 (1.00–6.00)	63	3.00 (1.00–5.00)
Generalized anxiety	88	4.00 (2.00–6.00)	77	3.50 (2.00–5.50)	63	4.00 (2.00–5.00)
Total score	86	21.00 (11.75–34.00) 0-88	76	18.50 (9.25–29.18) 0-64	63	17.00 (9.00–25.00) 0-52
SDSC						
Initiating and maintaining sleep	96	12.05 (10.10–15.00)	79	12.00 (10.00–14.00)	66	12.19 (10.00–15.00)
Sleep breathing	96	3.00 (3.00–4.00)	79	3.00 (3.00–4.00)	66	3.00 (3.00–3.55)
Arousal/nightmares	96	3.00 (3.00–4.00)	79	3.00 (3.00–4.00)	66	3.00 (3.00–4.00)
Sleep-wake transition	96	9.00 (7.00–10.75)	79	8.00 (7.00–10.00)	66	9.00 (7.00–12.00)
Excessive somnolence	96	7.50 (6.00–9.75)	79	7.00 (6.00–9.00)	66	7.00 (6.00–9.00)
Sleep hyperhidrosis	96	2.00 (2.00–4.00)	79	2.00 (2.00–4.00)	66	2.00 (2.00–4.00)
Total score	96	40.00 (35.00–45.88) 28-67	79	39.00 (34.00–41.00) 26-66	66	40.28 (34.00–45.00) 28-80

Note: Frequencies, medians and interquartile ranges, and minimums and maximums the the outcome measures at the different time points.

Friedman's tests also showed differences between time points for the SCAS Panic-agoraphobia (χ2(2) = 6.270, *p* = .044, n = 55) and the SDSC Arousal subscales (χ2(2) = 7.055, *p* = .029, n = 62). However, differences between time points were not statistically significant after Bonferroni adjustments (unadjusted significance for SCAS Panic-agoraphobia subscale .036 from baseline to follow-up). No differences between time points were found for any other measures.

### 3.4 Interaction of time and HCC on behavior

Two-way mixed ANOVAs were performed to analyze the effects of time (baseline, post-GCBT, and follow-up) and HCC (increase/decrease from baseline to follow-up) on CBCL score changes over the course of the study. Mauchly's test of sphericity indicated that the assumption of sphericity was met for the two-way interaction for CBCL-Int χ^2^(2) = 2.49, *p* = .288, CBCL-Ext χ^2^(2) = 3.42, *p* = .181, and CBCL-Tot χ^2^(2) = 3.59, *p* = .166. There were no interactions between time and HCC on CBCL-Int, CBCL-Ext, or CBCL-Tot scores. Main effects analyses showed that time had an effect on CBCL-Int, CBCL-Ext, and CBCL-Tot scores, while HCC did not (Table 5). Based on the results, it does not seem likely that HCC change mediated or had an overall impact on the effect of time on CBCL scores.

**Table 5:** Two-way Mixed Anova results.

Measure	Variables	df	*F*	*p*	η^2^
CBCL-Int	Time x HCC	2.98	1.10	.34	.022
	Time	2.98	8.14	<.001	.142
	HCC	1.49	0.63	.43	.013
CBCL-Ext	Time x HCC	2.98	0.01	.99	<.001
	Time	2.98	12.74	<.001	.206
	HCC	1.49	0.03	.86	.001
CBCL-Tot	Time x HCC	2.98	0.21	.81	.004
	Time	2.98	15.80	<.001	.244
	HCC	1.49	0.06	.81	.001

Note. Abbreviations: df, degrees of freedom; *F,* F-ratio, η^2^, Eta squared.

## 4 Discussion

Dysregulation of the HPA axis has been demonstrated in children with various psychiatric disorders [15,22,35,42,68]. Although the use of HCC as a biomarker of chronic stress in children is becoming common, there is limited data on HCC levels in childhood psychopathology [11,24,40,51]. Furthermore, the association of HCC with behavior and sleep disturbance symptoms has not, to the authors’ knowledge, been previously studied in clinical child psychiatric samples. Psychosocial interventions have been suggested to promote HPA axis regulation in children possibly contributing to improved mental health [57]. However, to our knowledge, HCC has not been used to assess the potential biological implications of CBT in children with psychopathology. Research evidence is important for evaluating intervention effectiveness and planning treatment considering biological mechanisms potentially associated with symptomatology.

The current study found no cross-sectional associations between HCC and behavior or sleep disturbance symptoms in a sample of 6–12-year-old child psychiatric outpatients with a variety of clinician-diagnosed psychiatric disorders presenting with both internalizing and externalizing behavior symptoms and sleep disturbance symptoms based on parent and/or teacher ratings. Hair cortisol appeared to be unrelated to the participants’ psychiatric diagnostic grouping, including depression/anxiety, ADHD, or other diagnoses. Furthermore, variations in HCC levels from baseline to follow-up were not associated with behavior symptom change after GCBT. Our findings open new avenues of research on the association between HCC and symptomatology in children and provide novel data on the utilization of HCC in exploring the effectiveness of CBT in child psychopathology.

The median baseline HCC level in our sample appeared elevated (5.33 pg/mg) compared to the age-adjusted HCC reference ranges for children of the same age (1.55 pg/mg-2.38 pg/mg for 6–12-year-olds) presented in the first LC-MS/MS-based study on healthy children aged 0–18 years [17]. The results may suggest chronic stress in our sample [39], providing support for the findings by Ref. [11] of elevated HCC in children with various psychiatric disorders. Presumably, children referred to GCBT at child psychiatric outpatient clinics would have experienced considerable stress in the preceding period. However, standardized norms for HCC have not yet been established, which makes it difficult to reliably determine stress thresholds. Furthermore, it should be noted that different laboratory techniques used to measure HCC make it difficult to compare HCC levels between study samples. For example, liquid chromatography-based analyses, used in our study, have yielded lower HCC concentrations than immunoassay protocols [59], which have so far been more commonly used in studies involving children [27].

Age and gender were the only variables correlated with HCC in our study, which was analyzed at two different time points. However, findings were inconsistent across time points preventing strong conclusions and further contributing to the inconclusive evidence on the relationship of HCC with age and gender [27,59]. The negative cross-sectional relationship between age and HCC at baseline suggested lower rates of HCC with age, inconsistent with the slow, upward trend in HCC in children aged 6–12 years reported in the study by Ref. [17]. Cross-sectional relationships between gender and HCC at post-GCBT partially support previous studies reporting findings of higher HCC levels in boys [27,64]. However, age and gender were not found to predict HCC. Although baseline HCC was not associated with behavior or sleep disturbance symptoms, child- and parent-rated internalizing symptoms and parent-rated total problem behavior symptoms were positively correlated with sleep disturbance symptoms, consistent with previous findings of associations between behavior and sleep disturbance symptoms in child psychiatric samples (e.g. Ref. [32]. Then again, externalizing problems were not correlated with sleep disturbance symptoms in our sample. The finding was unexpected given that sleep disturbances have been found to be associated with both internalizing and externalizing problems in youth with ADHD [18], and nearly 60 % of our sample had an ADHD diagnosis. However, a more detailed assessment of the relationship between behavior and sleep disturbance symptoms was beyond the scope of this study.

In addition, consistent with earlier research on the relationship between internalizing and externalizing symptoms [2], children's self-reported internalizing symptoms were correlated with parent-reported externalizing symptoms, and scores on internalizing and externalizing problems were correlated with each other in both parent and teacher ratings. Whereas both parents and teachers reported borderline to clinical range internalizing behaviors and clinical range externalizing and total problem behaviors, correlations between parent and teacher ratings were only found for externalizing problem scores. Our results are in accordance with findings of higher parent-teacher agreement for externalizing than internalizing problems [13]. Although child and parent ratings of internalizing symptoms were positively correlated, children's self-reported internalizing symptoms did not meet the threshold for elevated anxiety. Whereas research has shown that children as young as six years old may provide reliable and valid reports of their health [53], the nature of psychiatric disorders in our sample, particularly the proportion of ADHD diagnoses (58.5 %), may have a bearing on the findings. In a study by Ref. [63] on children with ADHD, parent-reported anxiety symptoms were associated with clinician ratings of comorbid anxiety, whereas children's self-reported symptoms were not. Overall, the observed correlations between child- and parent-reported symptoms were weak to moderate, while those observed among parent- and teacher-rated symptoms were moderate to strong. However, the magnitude of the correlations should be viewed with caution, as they were not adjusted for potential confounding factors. Lastly, the negative cross-sectional correlation of externalizing and total behavioral symptoms with age does not reflect development of symptoms in the children in our sample. There is evidence of persisting or increasing trajectories for externalizing and total behavior symptoms (e.g. Refs. [10,46], a more likely trend in clinical child psychiatric samples.

No changes in HCC levels were observed during the study suggesting that treatment may not have led to changes in cortisol production over time. The median HCC values of the participants oscillated slightly but not statistically significantly between time points. Changes during GCBT were observed only for parent-rated behavior symptoms. The level of symptom severity decreased for both internalizing and externalizing problems, with a drop in total problem behavior symptoms from the clinical level to normal variation. Long-term changes were prominent for externalizing and total problem symptoms with a shift in symptoms from the clinical range to the borderline range from baseline to follow-up. Our findings are consistent with those of a previous study from the same group [9] that found GCBT beneficial in reducing parent-rated behavior symptoms in a similar clinical sample of children with psychiatric disorders adding to the limited data on the immediate and long-term effects of CBT in children with co-occurring internalizing and externalizing symptoms (e.g. Ref. [37]). In the study by Ref. [9], the immediate effects of GCBT were compared with individualized treatment as usual delivered by mental health specialists. The adjustment for multiple comparisons and a slightly smaller sample size in the analyses of the present study may have contributed to the absence of a long-term effect for CBCL-Int, as observed in the previous study. Contrary to expectations (e.g. Ref. [56], no improvements were observed for self-reported internalizing symptoms following GCBT, which may be related to the pronounced representation of ADHD in our sample population [63]. A decreasing trend over the course of the study was found only for self-reported panic-agoraphobia symptoms. Unlike the previous study from the same group [9], the current study did not show reductions in teacher-rated internalizing or total problem behavior symptoms. The results of the previous study were based on symptom measures that were returned twice (before and after GCBT), while the results of the present study were based on symptom measures that were returned three times (before and after GCBT and at follow-up). As a result, the dataset was smaller due to a higher proportion of unreturned measures at follow-up, which in turn may have reduced statistical power. No reduction in parent-rated sleep disturbance symptoms was observed in the different sleep categories, with total scores remaining at or above the sleep disturbance threshold across time points. However, our longitudinal comparisons did not account for the potential variation in the nature and biological basis of sleep problems across psychiatric disorders [5]. Our findings complement the nascent data on parent-rated sleep-related outcomes after other than sleep-oriented CBT treatments, such as CBT for anxiety [3]. The review by Ref. [3] showed preliminary signs of CBT benefits in parent-reported bedtime resistance and sleep anxiety, that were not evaluated in our study.

Further analysis in the present study suggested that time had a beneficial effect on parent-rated behavior symptoms. Reductions in behavior symptoms over time appeared to be independent of any HCC variability from baseline to follow-up. Since improvements in parent-rated behavior occurred namely during GCBT rather than during follow-up, which was a longer period, the change may be more likely interpreted as a treatment-related rather than a time or age-related improvement. Treatment effects are supported by evidence from parent ratings that high levels of internalizing and externalizing problems tend to persist or increase across childhood (e.g. Refs. [21,46]. Our results did not provide support for the findings of [29] that an increase in HCC with treatment was associated with a reduction in behavior problems. They proposed that the increase in HCC may have possibly been related to the activation of coping mechanisms. Our study design, however, differed from their study in terms of intervention type, target population, and age group, and we looked at change as a categorical rather than continuous variable. Interestingly, nonetheless, HCC was at its highest at follow-up in our sample.

### 4.1 Limitations

To the authors’ knowledge, this is the first study to investigate HCC longitudinally in children with psychiatric disorders. However, there are several limitations. First, the increasing amount of missing data towards the end of the study due to unreturned measures may have resulted in some loss of statistical power. Additionally, whereas our sample's heterogeneity, with over 70 % psychiatric comorbidity, reflects real-world pediatric clinical diversity, a larger sample size would have been needed to confidently identify or rule out potential findings. Second, the study did not control for medication use, potentially obscuring relationships between HCC and symptoms. Hair cortisol [62] and sleep [61] may be affected by stimulants, the primary medications used by our participants. Additionally, anthropometric factors, potentially associated with HCC levels [41], were not accounted for. The HCC levels in our study sample varied somewhat, albeit statistically insignificantly, over time for which the underlying factors remained unclear. A larger data set may have allowed the examination of potentially differing trends in HCC over time. Finally, due to the lack of a control group in the present study, interpretations of the effects of GCBT on HCC are mainly indicative. As treatment took place in clinical settings, it was not possible to employ a non-treatment control group. Nevertheless, our results provide important qualitative data on the longitudinal HCC levels in children with psychiatric disorders.

## 5 Conclusions

Our findings suggested signs of elevated HCC in children with a range of clinician-diagnosed mixed psychiatric disorders. No cross-sectional associations were found between HCC and behavior or sleep disturbance symptoms, whereas behavior and sleep disturbance symptoms showed mutual correlations. Additionally, HCC did not seem to be related to the type of psychiatric disorder in children. There were no changes in HCC levels with GCBT. Furthermore, variations in HCC levels over the duration of the study did not appear to be associated with behavior symptom relief following GCBT. While GCBT may reduce behavioral symptoms, these improvements may not be reflected in changes in HCC. Our findings suggest that while HCC may be an indicator of chronic stress in children with psychiatric disorders, it might not be a methodologically relevant biomarker of behavior or sleep disturbance symptoms in this sample population. However, further investigation into HCC and its relationship to psychiatric symptoms in children, particularly those with multiple psychiatric conditions, is warranted.

## Funding

This work was supported by the 10.13039/501100003125Finnish Cultural Foundation (grant numbers 00170137 and 00230247); the 10.13039/501100012691Olvi Foundation (grant number 201720207); New Children's Hospital, the Pediatric Research Center; The 10.13039/501100005744Foundation for Pediatric Research (grant numbers 190098, 200132, 210159, and 220186); the 10.13039/100030827Niilo Helander Foundation (grant number 200005); the Otto A. Malm Foundation; the Waldemar von Frenckell Foundation; and the Yrjö Jahnsson Foundation (grant numbers 20217393 and 20237644). Open access funded by Helsinki University Library.

## Data availability statement

Restrictions apply to the availability of some or all data generated or analyzed during this study to preserve patient confidentiality. The corresponding author will on request detail the restrictions and any conditions under which access to some data may be provided. The corresponding author takes responsibility for the integrity of the data and the accuracy of the data analysis.

## CRediT authorship contribution statement

**Sarianna T.A. Barron-Linnankoski:** Conceptualization, Data curation, Formal analysis, Funding acquisition, Methodology, Project administration, Resources, Visualization, Writing – original draft, Writing – review & editing. **Hanna K. Raaska:** Conceptualization, Data curation, Funding acquisition, Methodology, Project administration, Resources, Writing – review & editing. **Paula H. Reiterä:** Formal analysis, Methodology, Writing – review & editing. **Marja R. Laasonen:** Conceptualization, Methodology, Supervision, Writing – review & editing. **Marko J. Elovainio:** Conceptualization, Methodology, Supervision, Writing – review & editing.

## Declaration of competing interest

The authors declare that they have no known competing financial interests or personal relationships that could have appeared to influence the work reported in this paper.
